# Madelung’s disease in a non-alcoholic Ethiopian male patient mistaken for obesity: a case report

**DOI:** 10.1186/s12902-021-00803-9

**Published:** 2021-07-03

**Authors:** Yared Zenebe Zewde

**Affiliations:** Department of Neurology, College of Health Sciences, Addis Ababa University, P. O. Box: 41690, Addis Ababa, Ethiopia

**Keywords:** Madelung’s disease, Lipomatosis, Hyperuricemia, Non-alcoholic, Ethiopian

## Abstract

**Background:**

Madelung’s disease (MD) is a rare disorder of fat storage characterized by the presence of diffuse, symmetrical deposition of subcutaneous fat around the neck, shoulder, arm, trunk and thigh. Although its cause is not fully understood, this benign condition is commonly presented among adult males with Mediterranean origin and history of alcohol abuse. Patients often presents with compression of vital structures, cosmetic disfigurement and associated psychosocial problems and systemic comorbidities. It is often under-recognized by physicians, possibly due to obliviousness of the condition and often misdiagnosed as obesity.

**Case presentation:**

We present a 65-year-old non-alcoholic black Ethiopian man, presented with a slowly growing body fat in his trunk and proximal limbs associated by multiple joint and back pain which got worse recently. He denied any history of chronic alcohol use. On examination, huge, bilateral, non-tender, soft, globular masses in his torso, shoulder, arm and thigh with bilateral breast enlargement. On investigation his biochemical profile was normal except hyperuricemia (10.6 mg/dl). Imaging of the cervical and lumbar vertebrae showed excess subcutaneous fat depositions with degenerative disc disease. Biopsy from the mass revealed non-encapsulated lipoma and he was diagnosed with type II MD. We treated his pain with supportive therapy and discharged in stable condition. The patient deferred surgical treatment.

**Conclusions:**

Madelung’s disease is often reported among white adult males with chronic alcoholism. However, our case reported a black man without the typical risk factor which was misdiagnosed as obesity. Hence, clinicians should be aware of MD and need to consider it in their differential diagnosis when encountered with a patient having progressive centripetal fat deposition with or without a history of alcoholism and systemic comorbidities. As early detection of this disorder helps to avoid diagnostic delays and prevent complications through timely interventions which will in turn improves patient quality of life.

## Background

Madelung’s disease (MD), also known as Launois-Bensaude syndrome, benign multiple symmetrical lipomatosis, and symmetrical adenolipomatosis, is a rare disorder characterized by deposition of non-capsulated fat tissues in typical locations -neck, trunk, and proximal limbs symmetrically. This usually leads to a disfiguring body but huge lipomas around the neck might be fatal from upper airway obstruction and cardiac complications[1].

Although the exact pathogenesis of MD is not clear yet, around 90 % of patients with MD had a history of chronic alcohol use. The disease is more common in a middle-aged man of Mediterranean or eastern European descent [2]. Herein, we present a case of 65 years-old Ethiopian male patient, without alcoholism history, presented with a complaint of multiple joint and back pain and progressive fat deposition in his trunk and proximal limb which was misdiagnosed as obesity in the past.

## Case presentation

A 65-year-old right-handed black Ethiopian male referred to the outpatient neurology clinic in Tikur Anbessa Specialized Hospital with a complaint of diffuse, symmetrical body fat deposition which was started 15 years back as belly fat and slowly grows to involve his anterior chest with bilateral breast enlargement. The mass continued to gradually increase until it attains the current size which now involves his torso, both upper arms and thighs. The mass spares his face and distal limbs. Since the past six months, he experienced deep aching pain in his knees and ankles bilaterally and recently the joint pain got worse with new onset low back pain. For the above complaint he visited a nearby clinic and his symptoms were attributed to his excess body weight (114 kg) at that time and treated with oral analgesic and advised to control his weight through diet and regular exercise. However, all of his symptoms persisted with worsening of back pain and walking difficulty. Which made him homebound and need another person assistance to stand and walk around the house. Otherwise, he did not have breathing or swallowing difficulty, abdominal pain or distal limb sensory or motor abnormalities. He denied any history of alcohol abuse, tobacco or illicit drug use but occasionally he consumed 1 to 2 beer in social events and holidays. He usually found it difficult to find a cloth that fits him otherwise he was relatively healthy. He lost his truck driving job since the recent worsening of symptoms and become financially dependent on his wife. He was married and a father of three boys. All of his siblings were alive and healthy except his older sister, who was hypertensive. His family history was negative for similar condition.

**Fig. 1:**
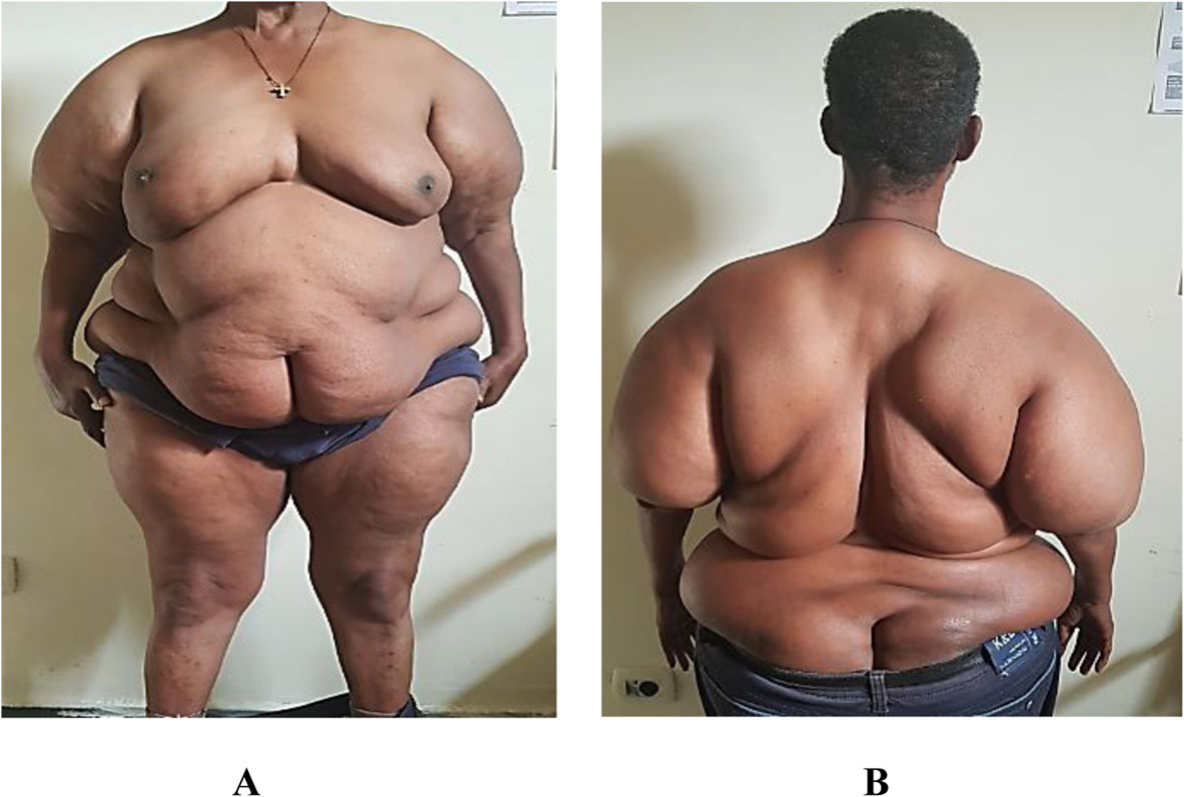
**A ***Anterior view* - Bilateral symmetrical breast enlargement with central fat deposition. **B** *Posterior view* – thick skin folds with huge fat deposits in posterior neck & back

**Fig. 2:**
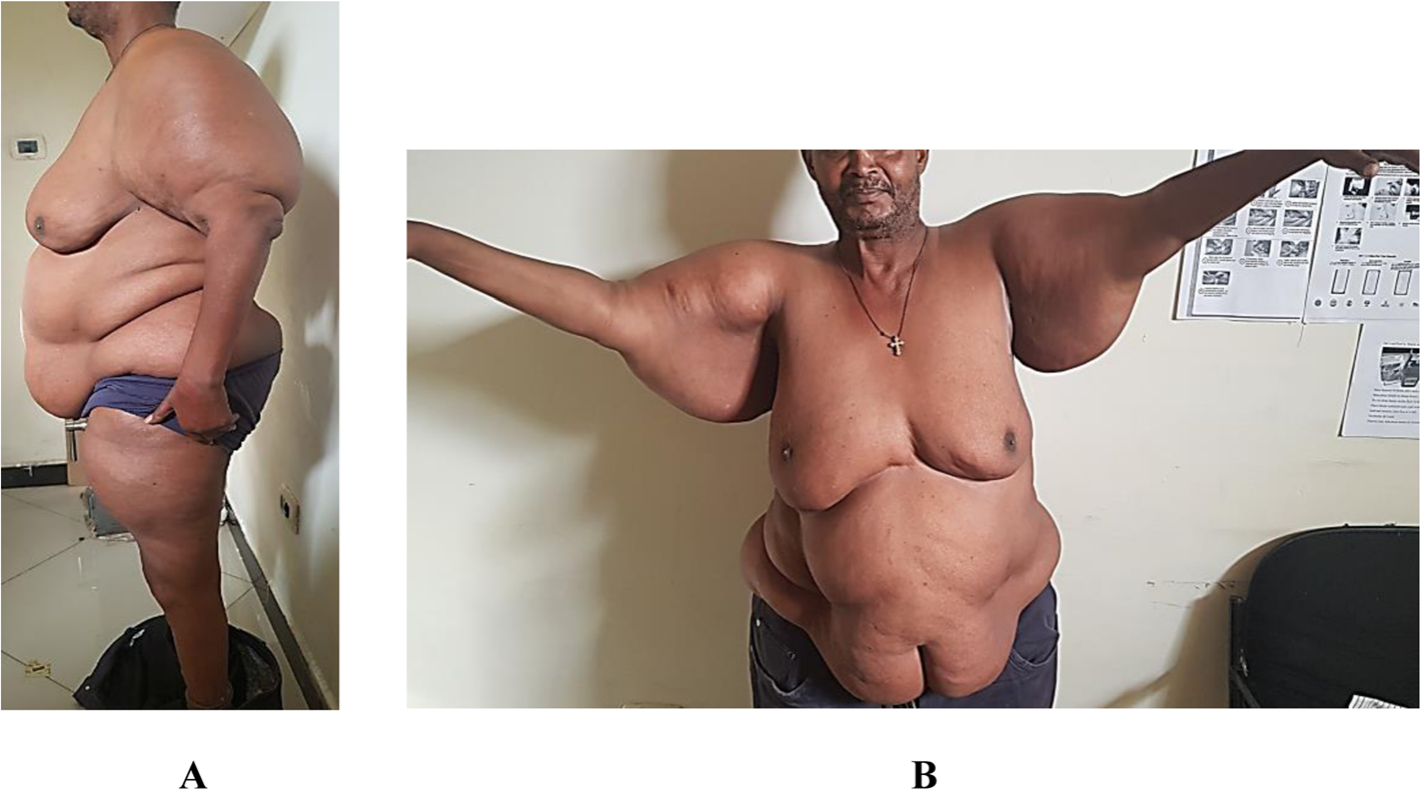
**A** *Lateral view*- Disproportional fat deposit in proximal arm, thigh and waist region with normal appearing forearm and shin. **B** *Anterior view* – Huge subcutaneous fat suspended out from an outstretched arm

**Fig. 3:**
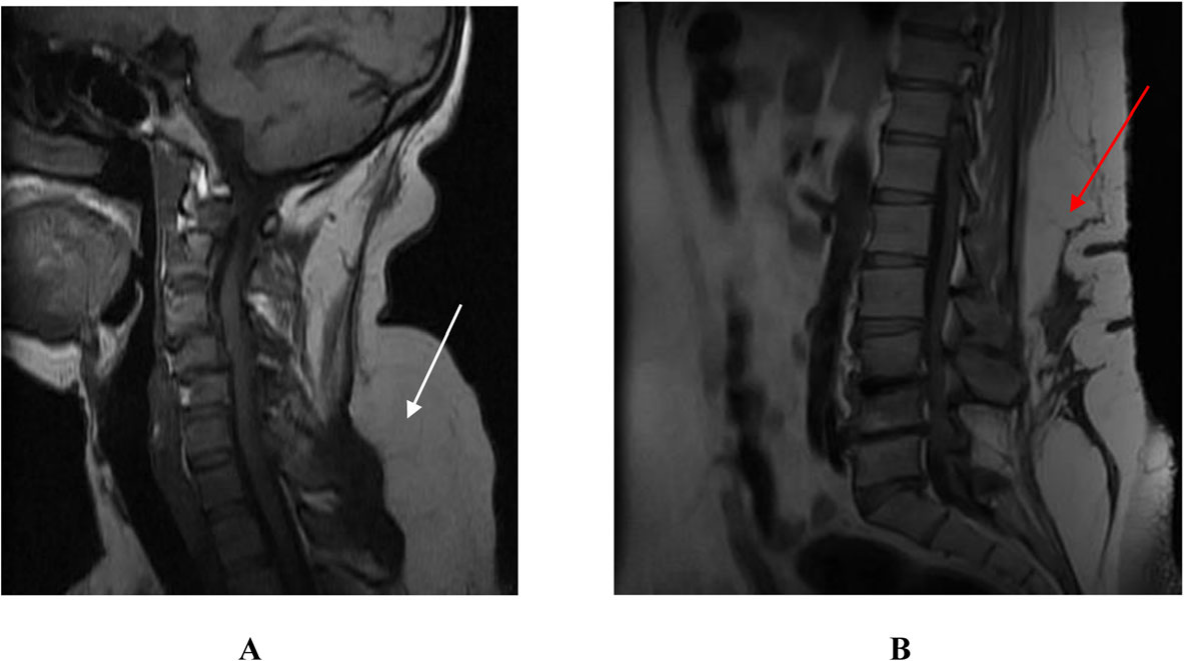
Sagittal T1-Weighted MRI of the cervical (**A**) and lumbar (**B**) spine shows huge subcutaneous fat depositions in the posterior neck (white arrow) and the lower back (red arrow) with multilevel degenerative disc disease

On physical examination, his body mass index was 39.6 kg/m^2^ (weight − 113 kg, height − 1.69 m) otherwise vital signs were normal. There was diffuse bilateral symmetrical, non-tender, soft swelling of his trunk, shoulder, upper arms and thighs with bilateral breast enlargements. [Figure 1(A)]. Also, he had thick and redundant skin folds with huge lipomas in his back torso sparing the neck [Fig. 1(B)], and disproportional enlargement of both arms and thighs as compared to a normal appearing forearm and shine [Fig. 2(A) & (B)]. On neurologic exam, straight leg raise test was difficult to perform because of the central mass. He had mild lumbar vertebrae tenderness. The motor and sensory exams were normal. No joint swelling, tenderness, or crepitation detected. The rest of his examination was non-revealing. Table 1 summarized the baseline laboratory findings where most of the results were in the normal range except hyperuricemia (10.6 mg/dl). Magnetic resonance imaging (MRI) of the cervical [Fig. 3(A)] and lumbar [Fig. 3(B)] vertebrae showed diffuse subcutaneous fat deposits in the posterior neck and lower back with multilevel degenerative disc prolapse and osteophytic changes. Biopsy from the mass revealed non-capsulated sheets of mature adipocytes with modest fibrous infiltration which is consistent with the pathologic features of benign lipomatosis.

**Table 1:** Summary of the laboratory findings for our MD patient with lab reference value

Laboratory test	Result	Normal reference value
**Fasting blood sugar**	92	<100 mg/dl
**Glycated hemoglobin (HbA1C%)**	4.8	4 - 5.6 %
**Lipid panel**		
Total cholesterol	139	<200 mg/dl
Triglyceride	121	<150 mg/dl
LDL- Cholesterol	77	<130 mg/dl
HDL- Cholesterol	42	40-60 mg/dl
**Liver function test**		
Alanine Aminotransferase/ALT	17	0-40 IU/L
Aspartate Aminotransferase/AST	15	0-40 IU/L
Alkaline Phosphatase	206	65-270 IU/L
Total Bilirubin	0.7	0-1.0 mg/dl
Direct Bilirubin	0.15	0-0.2 mg/dl
**Thyroid function test**		
Free T3	2.32	2.1 – 4.4 pg/ml
Free T4	1.29	0.8 – 2.7 ng/dl
Thyroid stimulating hormone	1.52	0.25 – 5 micIU/ml
**Renal function test**		
Blood urea nitrogen (BUN)	16	15 – 50 mg/dl
Creatin	0.91	0.6-1.2 mg/dl
**Uric acid**	10.6	3.5 – 7.2 mg/dl
**Rheumatoid factor**	Negative	
**HIV serology**	Negative

The patent was diagnosed with type II Madelung’s disease and lumbar degenerative disc disease with and hyperuricemia. We started with on parenteral nonsteroidal anti-inflammatory (Diclofenac) and physical therapy. A week after starting the supportive therapy his symptoms subsided and discharged home with oral analgesics. Advised on lifestyle and dietary modifications to control the hyperuricemia including abstinence from alcohol, avoiding high glycemic foods and sugary drinks that will limit his total daily calorie intake between 800 and 1000 calories, adherence to low purine diets such as nuts, whole grains, vegetables and fruits rich in vitamin C and engaging in regularly physical activity with at least 30 min of brisk walking for 3–5 days per week. The patient refused surgical treatment. One month in his follow up, he lost 2 kg with good functional recovery but lipomatosis remined unchanged. He was kept on regular follow-up in the endocrine, rheumatology and neurology outpatient clinics.

## Discussion and conclusions

Madelung’s disease (MD) was first described by Benjamin Brodie in 1846 in London hospital and subsequently named by Otto Madelung in 1888 [3]. Later in 1898, Launois and Bensaude, characterized the distinct features of the disease and termed benign symmetrical lipomatosis/ Launois-Bensaude syndrome [4].

MD is a rare disorder with an incidence of 1 in 25,000 and often identified in people of European or Mediterranean descent. The disease is more common among middle-aged white man between the age of 30 and 60 years with a male-to-female ratio of 15:1 to 30:1 [5]. There were very few reports of MD among Asian and African American population possibly due to misdiagnosis as a case of obesity like our patient.

Although the cause of MD is not clear yet but it was hypothesized that alcohol induced dysfunction of beta-adrenergic receptors in adipose tissue of alcoholic patients may promote subcutaneous fat accumulation, while the hereditary form is associated with mitochondrial DNA mutation and abnormal lipogenesis [2, 6].

Based on clinical presentation, there were two types of MD. Type I presents with tumor-like masses around the neck and back, giving the patient a ‘‘buffalo hump” and type II MD resulting in “pseudo-athletic appearance” with diffuse fat deposition over the trunk and proximal part of limbs [7]. Our case was diagnosed with type II MD based on the areas involved.

Systemic comorbidities such as epilepsy, diabetes, Cushing’s disease, primary hypothyroidism, macrocytic anemia, peripheral neuropathy, and some malignancies have been associated with MD [8]. Despite extensive workup none of these conditions identified in our patient.

Diagnosis is mainly clinical however, imaging studies (CT, MRI and ultrasonography) can be helpful to assess its extension when surgical excision is planned. Biopsy may help to confirm the diagnosis and exclude its mimickers such as angiolipoma, neurofibroma, lipodystrophy, and liposarcoma [2, 8].

There is no definite treatment for MD but nonsurgical and surgical options were used in the real world with variable success rate. A recent systematic review of 52 published articles on MD treatment showed that 91.5 % of the patients were treated surgically with lipectomy or liposuction or in combination. Lipectomy was the commonest procedure (89.6 %) preferred for severe cases having multiple mass and airway compression. It has an advantage of better clinical outcome with 5 % recurrence. In contrary, liposuction is a simple, less invasive procedures with better cosmetic outcomes in less severe, diffuse lipomas, however this was canceled out by high recurrence rate [9]. Non-surgical options such as abstinence for those with alcohol history, weight control, and drug therapy with Beta-adrenergic agonists and fibrates can be used [2, 6, 7].

New techniques such as ultrasound-assisted liposuction (UAL) [10], tunneling technique [11] and power assisted liposuction [12] emerging as a safe and effective therapeutic option. Although most cases of Madelung’s disease have benign prognosis, a rare case of malignant transformation was reported in the literature [13], which necessitates early identification.

In conclusion, to the best of our knowledge, this is the first case of Madelung’s disease reported from Ethiopia and possibly in Africa. Clinicians need to consider MD in their differential diagnosis when encountered with a patient with a slowing growing body fat with a centripetal distribution that can be complicated by metabolic and neurologic conditions. As early diagnosis and timely intervention might prevent serious complications and improves patient quality of life.

## Data Availability

All data generated during this report are included in this manuscript.

## Abbreviations

CT: Computed tomography
HIV: Human immunodeficiency virus
Kg: Kilogram
MD: Madelung’s disease
MRI: Magnetic resonance imaging
NSAID: Non-steroidal anti-inflammatory drugs
UAL: Ultrasound-assisted liposuction
